# Special FDA designations for drug development: orphan, fast track, accelerated approval, priority review, and breakthrough therapy

**DOI:** 10.1007/s10198-023-01639-x

**Published:** 2023-11-14

**Authors:** Daniel Tobias Michaeli, Thomas Michaeli, Sebastian Albers, Tobias Boch, Julia Caroline Michaeli

**Affiliations:** 1 Department of Medical Oncology, National Center for Tumor Diseases, Heidelberg University Hospital, Im Neuenheimer Feld 460, 69120 Heidelberg, Germany; 2 TUM School of Management, Technical University of Munich, Munich, Germany; 3 Department of Personalized Oncology, University Hospital Mannheim, Heidelberg University, Mannheim, Germany; 4 DKFZ-Hector Cancer Institute at the University Medical Center Mannheim, Mannheim, Germany; 5 Division of Personalized Medical Oncology, German Cancer Research Center (DKFZ), Heidelberg, Germany; 6 Department of Orthopaedics and Sport Orthopaedics, School of Medicine, Klinikum Rechts Der Isar, Technical University of Munich, Munich, Germany; 7 Department of Obstetrics and Gynaecology, LMU University Hospital, LMU Munich, Germany

**Keywords:** Orphan designation, Fast track, Accelerated approval, Priority review, Breakthrough therapy, Clinical trial, Innovation, US food and drug administration, European medicines agency, Drug development, Special designation, Safety, Efficacy, Healthcare policy, Pharmaceutical policy, Drug price, I00, I1, I18, I13

## Abstract

**Background:**

Over the past decades, US Congress enabled the US Food and Drug Administration (FDA) to facilitate and expedite drug development for serious conditions filling unmet medical needs with five special designations and review pathways: orphan, fast track, accelerated approval, priority review, and breakthrough therapy.

**Objectives:**

This study reviews the FDA’s five special designations for drug development regarding their safety, efficacy/clinical benefit, clinical trials, innovation, economic incentives, development timelines, and price.

**Methods:**

We conducted a keyword search to identify studies analyzing the impact of the FDA's special designations (orphan, fast track, accelerated approval, priority review, and breakthrough therapy) on the safety, efficacy/clinical benefit, trials, innovativeness, economic incentives, development times, and pricing of new drugs. Results were summarized in a narrative overview.

**Results:**

Expedited approval reduces new drugs’ time to market. However, faster drug development and regulatory review are associated with more unrecognized adverse events and post-marketing safety revisions. Clinical trials supporting special FDA approvals frequently use small, non-randomized, open-label designs. Required post-approval trials to monitor unknown adverse events are often delayed or not even initiated. Evidence suggests that drugs approved under special review pathways, marketed as “*breakthroughs*”, are more innovative and deliver a higher clinical benefit than those receiving standard FDA approval. Special designations are an economically viable strategy for investors and pharmaceutical companies to develop drugs for rare diseases with unmet medical needs, due to financial incentives, expedited development timelines, higher clinical trial success rates, alongside greater prices. Nonetheless, patients, physicians, and insurers are concerned about spending money on drugs without a proven benefit or even on drugs that turn out to be ineffective. While European countries established performance- and financial-based managed entry agreements to account for this uncertainty in clinical trial evidence and cost-effectiveness, the pricing and reimbursement of these drugs remain largely unregulated in the US.

**Conclusion:**

Special FDA designations shorten clinical development and FDA approval times for new drugs treating rare and severe diseases with unmet medical needs. Special-designated drugs offer a greater clinical benefit to patients. However, physicians, patients, and insurers must be aware that special-designated drugs are often approved based on non-robust trials, associated with more unrecognized side effects, and sold for higher prices.

## Introduction

**Fig. 1:**
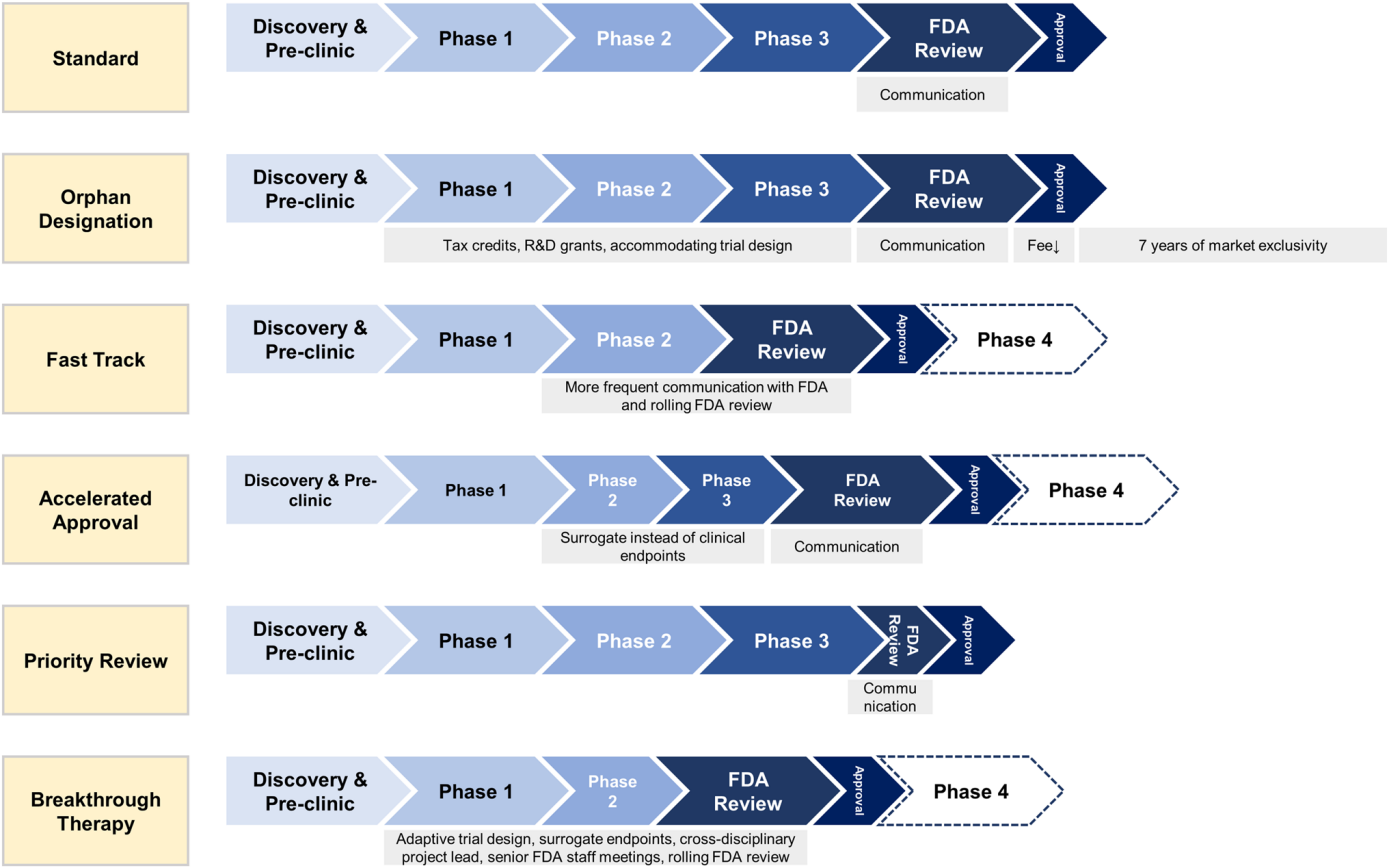
An overview of the FDA’s special review pathways and designations. *FDA* US food and drug administration, *R&D* research and development. Own illustration

In the US, drug development is regulated by the US Food and Drug Administration (FDA). The FDA grants special designations^1^ to expedite drug development and regulatory review for promising drugs treating diseases with unmet medical needs^2^ [2, 3]. To date, five special FDA review programs exist for cancer drugs^3^: orphan, fast track, accelerated approval, priority review, and breakthrough therapy designation (Fig. 1) [2, 3]. Even though the standards of the drug approval process—“safety and efficacy”—remained untouched since its inception in 1962, these special procedures offer more flexibility for companies to investigate drugs in disease-and therapy-tailored clinical trials and for the FDA to approve drugs based on a wider variety of efficacy measures [9].

The use and potential misuse of special FDA programs are widely debated in science, healthcare policy, and the general public [8–11]. While most scholars agree with the concept of directing research and development (R&D) efforts toward diseases with few treatment options and granting early access to innovative drugs (Box 1), there is an ongoing discourse about thereby created economic incentives and potentially adverse implications for patients [9, 10]. Expediting drug development naturally reduced the time to market; yet, faster drug development and regulatory review were identified to be associated with unrecognized adverse events and post-marketing safety revisions, e.g., withdrawals or warnings [12–15]. Consequently, the FDA often requires companies to monitor a drug’s adverse events after approval in post-marketing trials (phase 4) if its safety is uncertain [16, 17]; yet post-approval trials are frequently delayed or not even initiated [18, 19]. Meanwhile, no conclusive evidence exists that drugs approved under special review pathways are more effective than those approved under the standard FDA process [20–23]. All this may suggest that special review programs do not “coincide with patients’ interests”—scholars even accuse the pharmaceutical industry and US Congress of driving reforms in their interest under the preamble to benefit patients [24]. In addition, drugs approved under expedited review are quickly adapted to clinical routine [25], priced for a premium [20], and thereby consume significant financial resources which induces a great burden on the healthcare system [26, 27].

**Fig. 2:**
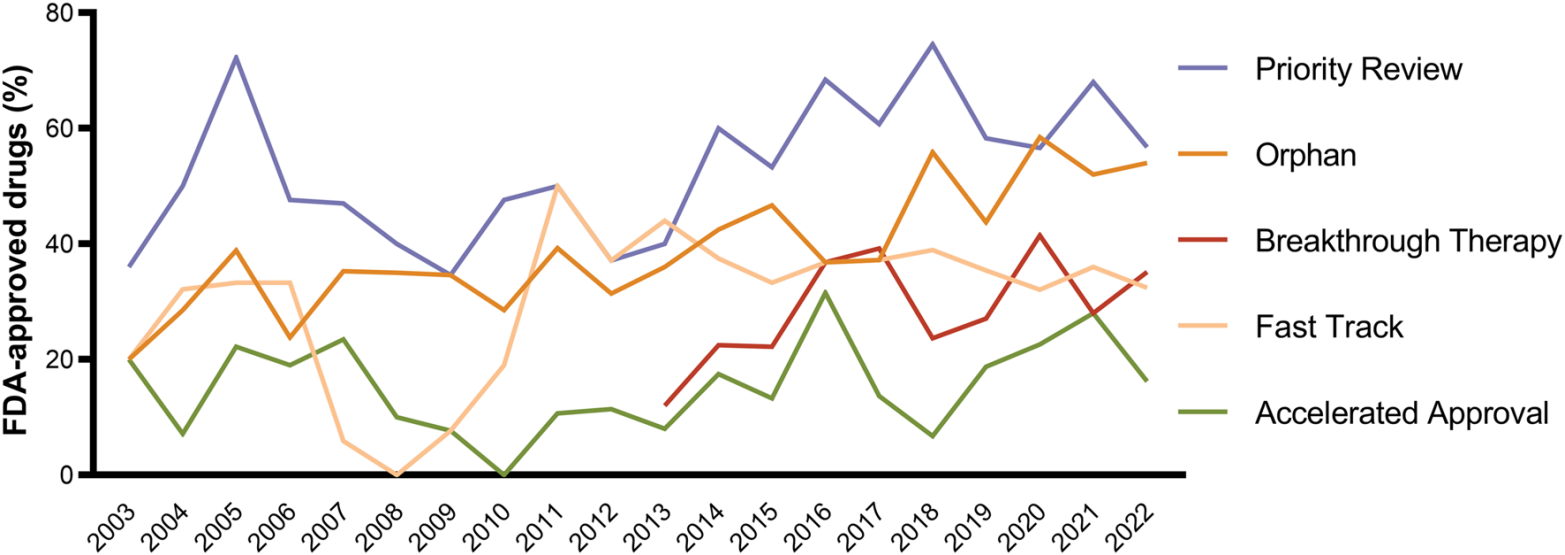
Drugs with special FDA approval from 2003 and 2022. *FDA* US food and drug administration. Own illustration

Figure 2 illustrates the share of FDA-approved drugs receiving each of the five special designations and review programs over the past 20 years. Two-thirds of new drugs received at least one special designation. Out of 666 new drug approvals, 367 (55%) received priority review, 279 (42%) orphan designation, 215 (32%) fast track, 124 (29%^4^) breakthrough therapy, and 103 (15%) accelerated approval. The share of drugs receiving the orphan designation increased from 20% in 2003 to 54% in 2022. Similarly, the breakthrough therapy designation gained in popularity after its introduction in 2012, with 12% of drugs receiving the designation in 2013 and 35% in 2022.

The purpose of this article is to review the FDA’s special approval pathways and designations regarding their safety, efficacy/clinical benefit, clinical trials, innovation, economic incentives, development timelines, and price. To identify relevant scientific literature on the FDA’s special designations and programs, we conducted a keyword search of the respective program names and “FDA” in PubMed. Results were summarized in a narrative overview. A brief summary of the FDA review and approval process can be found in Box 2.

Box 1. Push and pull policies to incentivize research and development of new drugs The recent coronavirus pandemic demonstrated that governments can incentivize drug development in areas of high unmet medical needs [133, 134]. R&D of new drugs can be stimulated through *push* and *pull* incentives [135–138].

**Push incentives **Push policies provide companies with a favorable environment to conduct research in basic technologies. These incentives may include public research funding through universities or governmental institutes, tax credits, research grants, and government-sponsored venture incubators and accelerators. For example, the bipartisan 21st Century Cures Act (2016) not only laid the foundation to facilitate data sharing with less bureaucracy in biomedical research [139], but also provided funding for specific therapeutic areas with unmet needs and for promising new technologies [140]. Oncology received $1.8 billion in funding with the Cancer Moonshot Initiative, neurology $1.5 billion with the Brain Research through Advancing Innovative Neurotechnologies Initiative, $1.5 billion with the Precision Medicine Initiative, and regenerative medicine $30 million [140].

**Pull incentives **On the other hand, policymakers can introduce legislature to pull companies’ R&D projects into a certain market [135–138]. These comprise awards, prolonged exclusivity periods, advanced purchasing agreements, higher drug prices, and expedited regulatory approval. Expedited regulatory approval takes a special role in these programs. Not only does it aim to incentivize the development of drugs for life-threatening diseases, but also hopes to bridge the “valley of death” between pre-clinical drug discovery and regulatory approval by increasing clinical trials’ probability of success through closer collaboration between pharmaceutical companies and regulators [2, 3].

Box 2. The FDA review and approval process The FDA evaluates a drug’s safety and efficacy based on the submitted New Drug Application (NDA) or Biologic License Application (BLA).^5^^,^ 6 The review process is structured into three broad steps, starting with an analysis of the underlying disease and currently available therapeutic options, a benefit and risk assessment based on the clinical evidence, and finally risk management considerations [141]. It is first necessary to review a disease’s incidence, prevalence, burden for society, mortality rates, pathology, and therapeutic options to provide a context for the benefits and risk assessment. This assessment considers the quality and quantity of all evidence in its totality. As such, the study design, outcome, side effects, inclusion and exclusion criteria, alongside its pharmacological properties are reviewed. Overall, the benefits of approving a drug should outweigh its risks and uncertainties arising from inconclusive evidence, missing data, adverse events, and not studied patient populations. In case of uncertain patient safety, the FDA may institute a risk evaluation and mitigation strategy (REMS) to monitor a drug’s side effects. Moreover, an expert advisory committee, which reviews the evidence on the considered drug and then provides a supportive recommendation to the FDA to guide decision-making, can be assembled. However, the recent approval of aducanumab in 2021, which was not recommended by the committee, shows that the FDA is not bound by its judgment [142]. The FDA currently has a 10-month time period to conduct this review and make a final approval decision. US Congress innovated the FDA’s review methods since its inception in 1962 to account for advances in medicine, biotechnological discovery, and drug development.

## Orphan designation

US Congress recognized that there is little economic incentive for companies to develop drugs for rare diseases.^7^ The patient population of diseases with a low incidence rate is too small for companies to offset the high R&D costs associated with bringing a new drug to market. An unaffordable price premium would otherwise be required for orphan drugs to be financially viable to develop. Moreover, pharmaceutical companies struggled to recruit skilled investigators, enroll the right patients, and fund large-scale phase 2 and 3 trials [28]. As a consequence, companies neglected rare diseases in their R&D efforts, resulting in large unmet medical needs—20–25 million citizens suffered from 5,000 rare diseases in the US by the 1980s [29]. US Congress sought to address these unmet medical needs by introducing the Orphan Drug Act (ODA) in 1983.^8^

The ODA comprised a bundle of push and pull policies to increase the number of drugs developed for rare diseases. Push policies offer tax credit (25% of clinical R&D costs),^9^ research grants for orphan R&D projects, and no NDA/BLA submission fee (which may amount to over $3 million), while a prolonged market exclusivity of 7 years pulls drug developments toward indications with a low prevalence. The ODA also bridges the period between IND and NDA approval by instituting that orphan drugs are eligible for closer collaboration with the FDA and permitting disease-tailored clinical trial designs. The FDA even encourages the use of “innovative clinical trial methods such as adaptive and seamless trial designs, modeling and simulations, and basket and umbrella trials” [33].

**Fig. 3:**
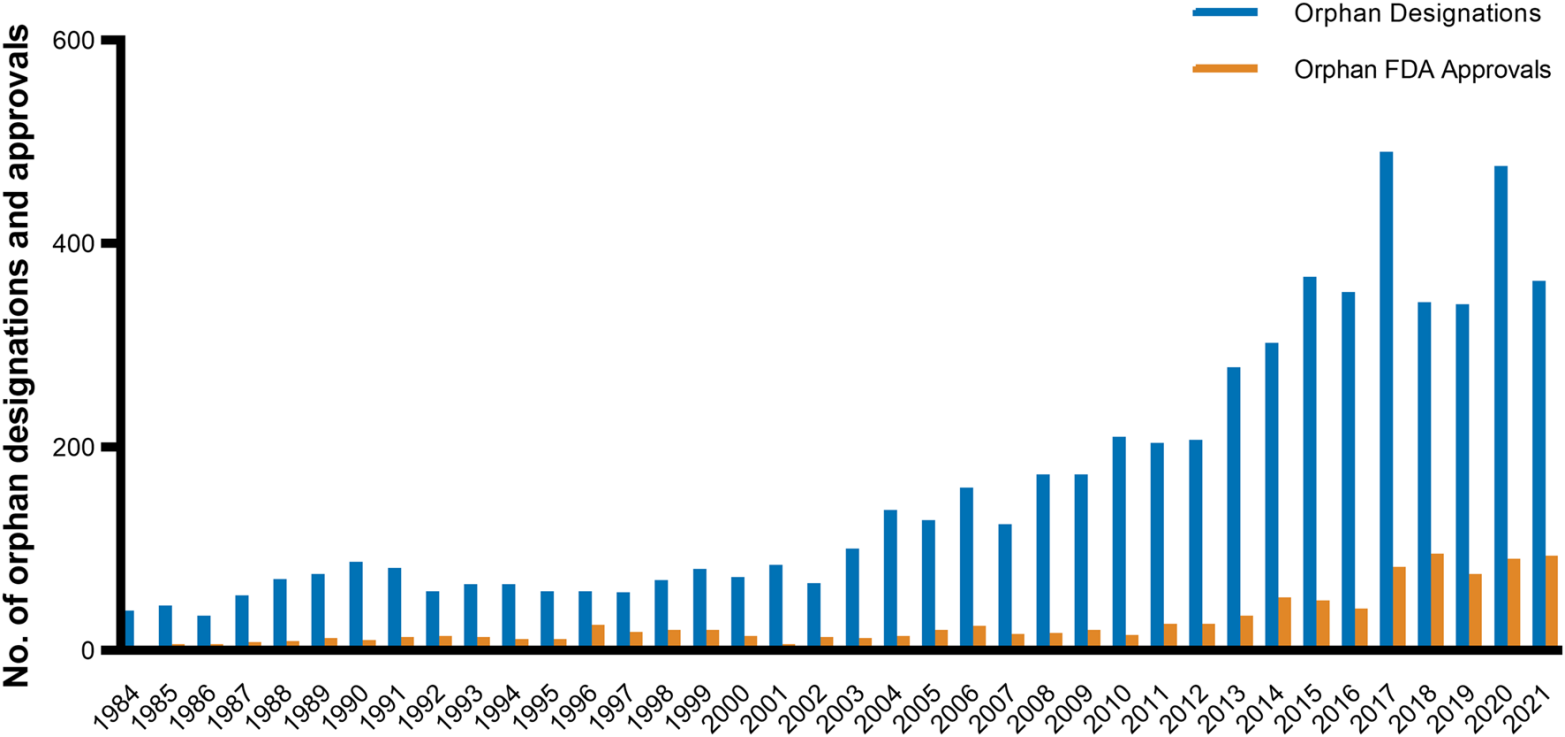
Orphan FDA designations and approvals from 1983 until 2021. *FDA* US food and drug administration. Own illustration

The success of the ODA was observed in the following decades (Fig. 3). From 1983 until the end of 2021, the FDA granted the orphan designation to 6,143 drugs. Of these, 1,033 received FDA approval. Until 2002, these new drugs collectively provided new treatments for around 11 million patients [29]. Nonetheless, orphan drugs were only developed for 15% of rare diseases and only 5% have an FDA-approved treatment [34]. The new approval route for orphan drugs comes with its challenges. A longitudinal study from 1999 to 2008 identified that out of 214 orphan drugs, 69% had changes in the FDA label’s safety section after approval, 15% being severe safety events, e.g., withdrawals, warnings, or suspensions [35]. Another study even observed that 87% of orphan drugs had serious safety events following approval by the European Medicines Agency (EMA) [31]. Part of the safety concerns surrounding orphan-designated drugs can be attributed to trial design. Orphan drug approvals were found to more frequently rely on non-randomized open-label single-arm trials, studying only half of the patient population enrolled in non-orphan trials [31, 36–38]. This is particularly concerning given that small, non-robust trials have a higher risk of bias and could thereby overstate treatment outcomes [39–41].

The efficacy and costs of orphan drugs are widely discussed. A review conducted by Onakpoya et al. suggests that orphan drugs provide significant treatment benefits to patients, especially considering that few other therapeutic options are available [31]. Building on Onakpoya et al., Chambers et al. reported a higher health gain, as measured by median incremental quality-adjusted life years (QALYs) gained, for orphan relative to non-orphan drugs (0.25 vs. 0.05 QALYs, *p* = 0.009) [42]. Accordingly, in a sample of 455 FDA-approved cancer indications, orphan relative to non-orphan indications extend progression-free survival (PFS) by 3.3 months and 2.8 months (*p* = 0.011), respectively [38]. Orphan indications were associated with greater improvements in PFS hazard ratios (0.51 vs. 0.64, *p* < 0.001) and tumor response rates in single-arm trials (51% vs. 33%, *p* < 0.001) [38]. Hwang et al. showed that orphan relative to non-orphan drugs have a 2.3 times higher likelihood to have a high therapeutic value for EMA, yet not FDA, approvals [43]. Nevertheless, Chambers et al.’s study of 218 indications also revealed greater costs ($47,652 vs. 2870, *p* < 0.001) which resulted in higher incremental cost-effectiveness ratios (ICER)^10^ for orphan drugs (276,288 vs. 100,360 $/QALY, *p* = 0.007) [42]. Similar to Chambers et al., higher mean monthly prices were reported for orphan than non-orphan cancer drugs in the US ($33,070 vs. $14,508, *p* = 0.020) [38]. Consequently, the availability of and access to orphan drugs is challenged by high prices [31, 32, 44]. Although there is heterogeneity in orphan drug pricing policies around the globe [32], prices are generally inversely correlated to disease prevalence [31, 45–48].

Several scholars estimated higher clinical trial success rates for orphan drugs. Based on a sample of 5820 indications under development from 2003 to 2011, Hay et al. estimated orphan indications have a higher than average chance to proceed from phase 1 to FDA approval (32.9% vs. 10.4%) [49]. Accordingly, Thomas et al., who assessed 7455 development programs between 2006 and 2015, observed an overall success rate of 25.3% for rare diseases compared to an average success rate of 9.6% for all development projects [50].^11^ In contrast, a more recent study of 21,143 development projects across 406,038 clinical trials from 2000 to 2015 found a lower probability of success for all orphan indications (6.2% vs. 13.8%), yet after excluding oncologic indications, the probability rises to 13.6% [51]. In a sample of 640 novel drugs that were developed between 1998 and 2008, Hwang et al. found a significantly higher probability for orphan than non-orphan drugs to be successful in clinical trials and receive FDA approval (adjusted odds ratio [AOR]: 2.26, 95% CI: 1.37 to 3.71) [52]. As a result of these higher success rates and fewer enrolled patients, Jayasundara et al. reported that the clinical development cost to bring a new drug to market is 29% lower for orphan relative to non-orphan drugs [53]. However, as a result of difficult and slow patient accrual in rare disease trials [54, 55], there is no significant difference in the clinical development time of orphan and non-orphan drugs [38].

The favorable economic incentives of orphan drugs are also recognized by pharmaceutical companies and investors [56]. Although Rooswinkel et al. found no significant difference between the valuation of 98 orphan and non-orphan mergers and acquisitions (M&As) between 2008 and 2012 [57], Michaeli et al. reported significantly higher late-stage company valuations based on a sample of 311 M&As between 2005 and 2020 [58, 59]. Combined with higher clinical trial success rates for orphan drugs, they calculated excess annual returns for investments in companies developing orphan drugs (46% vs. 12%, *p* < 0.001) [59]. Accordingly, an event study reported a 3.36% stock price increase following the announcement of the orphan designation [60]. A retrospective study of 86 orphan drug companies propensity matched to 258 controls, reported a significantly higher Tobin’s Q and market-to-book value [61]. This might be explained by the fact that orphan drug companies are simply more profitable than their peers, as measured by a 9.6% (95% CI: 0.6 to 18.7) higher return on assets and a 516% (95% CI: 19.8 to 1011) higher operating profit [61].

Previous studies highlighted that there are three distinct orphan subgroups of indications receiving the orphan designation [38, 62–65]. The orphan designation is granted to common diseases with orphan subgroups, e.g., “common orphans”, (prevalence > 200,000 US inhabitants), rare diseases (prevalence 6600–200,000 US inhabitants), and ultra-rare diseases (prevalence < 6,600 US inhabitants). Conducting clinical trials for ultra-rare is substantially more complex than conducting trials for rare or common diseases [38, 55]. Ultra-orphan drug development remains challenging as sponsors have trouble finding a sufficient number of patients, competent investigators, and specialized medical centers with adequate biotechnological infrastructure to administer their treatment. Guided by examples from the UK [66, 67], recent articles have, henceforth, proposed to introduce a distinct ultra-orphan designation [38, 68]. This ultra-orphan designation could entail greater tax credits (50%), a longer period of market exclusivity (10 years), more R&D grants, and greater collaboration between government institutes and industry to encourage drug development for ultra-rare diseases.

Moreover, scholars raised concerns about orphan drugs that are used to treat rare and common diseases—“partial orphans” [69]. These partial orphan drugs were found to be more frequently commercialized for the non-orphan than the orphan indication [69–71]. Partial orphans, thereby, frequently turn into top-selling blockbusters. In 2019, seven of the top ten grossing drugs were commercialized for an orphan and a non-orphan indication. Particularly drugs that are first approved for their orphan indications and then extend their marketing authorization to non-orphan indications (“orphan-first strategy”) are criticized for benefiting from high orphan price premiums [72–74]. Scholars proposed patient (e.g., < 200,000 US inhabitants) or revenue thresholds (e.g., < $200 million), indication-specific pricing, and indication-specific formularies to control the usage and expenditure on partial orphan drugs [68–71].

## Fast track

**Fig. 4:**
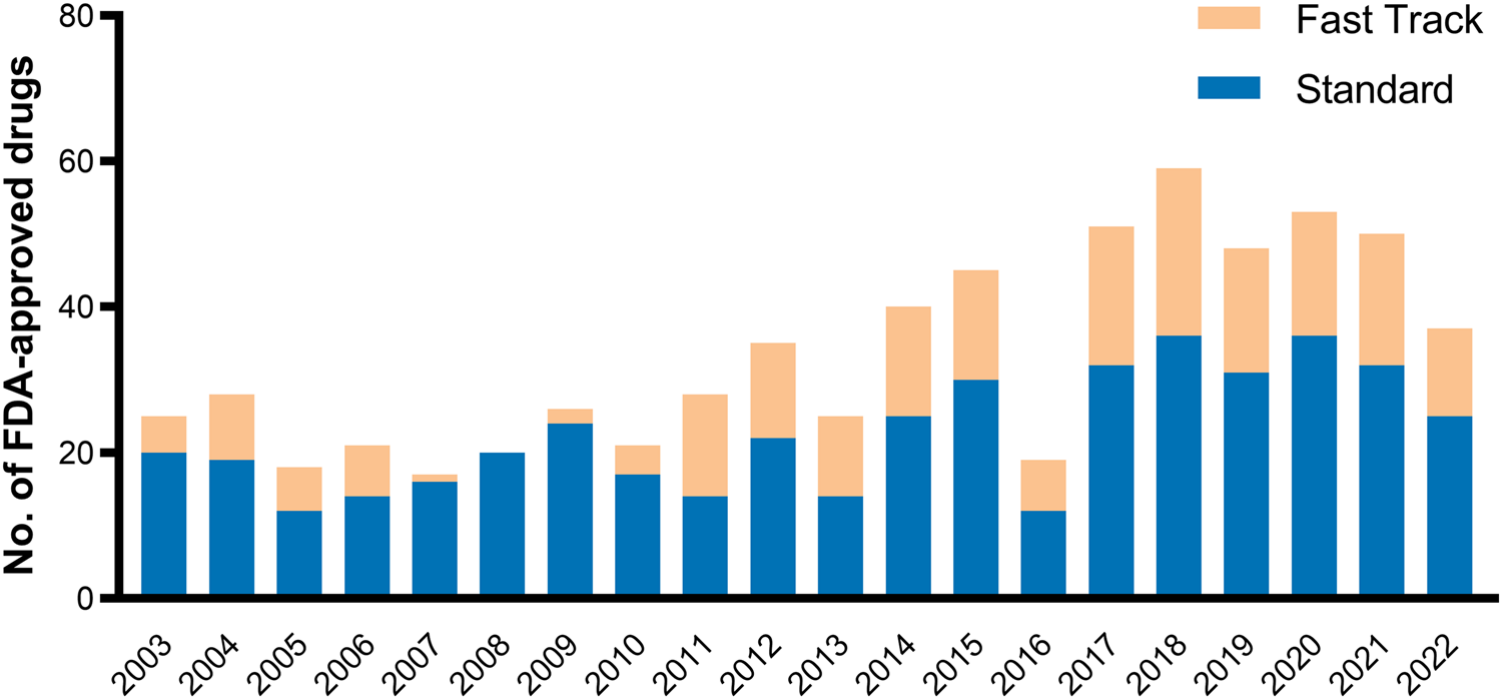
Drugs with standard and fast track FDA approval from 2003 until 2022. *FDA* US food and drug administration. Own illustration

In the 1980s, the acquired immunodeficiency syndrome (AIDS) was recognized as a pandemic around the globe [75]. New antiretroviral treatments were needed to quickly combat the emerging virus. In light of this emerging threat, the US, therefore, introduced the fast track program in 1988 to “facilitate the development, and expedite the review of drugs to treat serious conditions and fill an unmet medical need” [2, 10]. While the FDA recognizes that the classification of a condition as serious is a question of subjective judgment, it defines serious conditions by their survival, quality-of-life, or disease progression characteristics [2]. Under this definition, serious conditions include AIDS, dementia, cancer, heart failure, but also epilepsy, depression, and diabetes. In this context, the FDA interprets drugs treating a disease without other alternative treatments to fill an unmet medical need. In the case of available treatment alternatives, the unmet medical need could be filled with a new drug that is better than the existing therapy. This advantage can be demonstrated by superior efficacy, fewer side effects, earlier diagnosis resulting in better clinical outcomes, better and longer treatment adherence, or addressing future public health needs [2]. Under the new fast track process, the pharmaceutical company meets and closely collaborates with the FDA after a successful phase 1 trial to design a phase 2 trial which can build the basis for approval [76]. If successful, this phase 2, instead of phase 3, trial would, therefore, be sufficient to prove a drug’s safety and efficacy. It was argued that patients suffering from deliberating diseases require quicker access to promising drugs and are “willing to accept greater risks and uncertainty” [8]. Under this program, the FDA can continually review evidence generated from clinical trials (rolling review). However, the program also permits the FDA to demand a post-marketing trial in case of uncertain side effects, toxicity, or treatment outcomes. The clinical effects of the fast track program are more broadly discussed with the other special designations—here evidence on its economic implications is briefly reviewed. From 2003 until 2022, an average of 33% of new drugs were reviewed under the fast track program (Fig. 4).

The fast track designation sends a positive signal not only to patients, physicians, and insurers but also to investors. Chambers et al. reported that across 135 indications approved between 1999 and 2012, those with the fast track designation offered a median benefit of 0.254 incremental QALYs compared to 0.014 QALYs for those without the designation (*p* < 0.001) [77]. Coherently, Hwang et al. found fast track drugs have a threefold higher likelihood of being associated with a high therapeutic value [43]. As a result, drugs with a fast track designation may be able to demand higher prices before surpassing a country’s WTP threshold. The expedited development timelines alongside higher health benefits are valued by investors, as several event studies find companies with fast track designated treatments yield excess returns [78–81]. Yet, these excess returns seem to have diminished over time [80].

## Accelerated approval

Four years after the fast track program’s inception, in 1992, the US further expedited drug development by introducing the accelerated approval program.^12^ Similar to the fast track program, accelerated approval can be obtained for drugs treating a serious condition and hence fill an unmet clinical need [86]. Accelerated approval enables the FDA to judge a drug’s efficacy based on surrogate rather than clinical endpoints. Measuring a drug’s effect on patient survival may require a long trial duration and follow-up with many enrolled patients, especially for cancer types with high 5- and 10-year survival rates, e.g., prostate or breast cancer. In contrast, surrogate endpoints, such as tumor shrinkage or progression, can be more quickly observed and occur in most patients [87]. Therefore, the accelerated approval program expedited drug development by shortening clinical trial durations and enabling trial designs with fewer enrolled patients to measure surrogate endpoints. For example, Johnson et al. observed cancer drugs with accelerated approval reach the market 3.9 years faster than those with standard FDA approval [88]. Using data from 188 cancer indications with FDA approval, Chen et al. estimated that the use of the surrogate endpoints PFS and tumor response rate reduces clinical trial duration by 11 and 19 months, respectively [87].

**Fig. 5:**
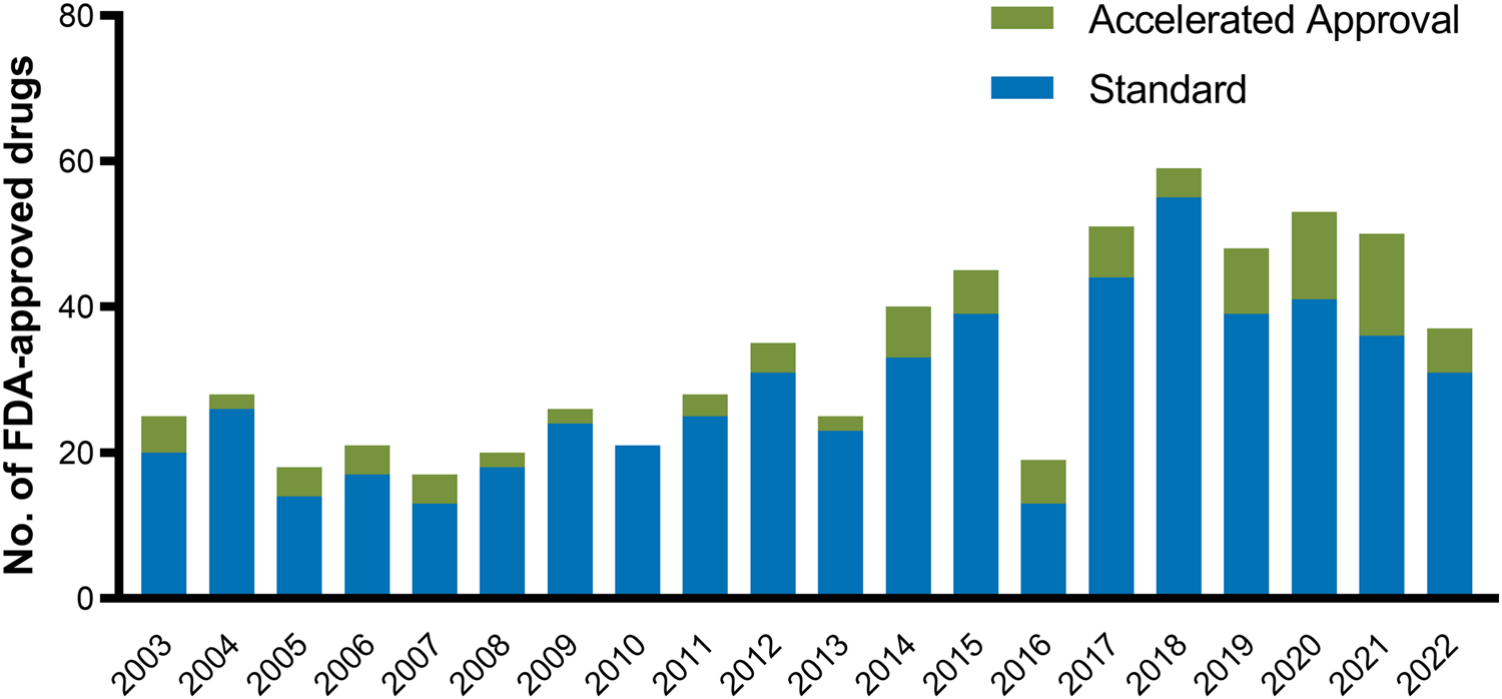
Drugs with standard and accelerated FDA approval from 2003 until 2022. FDA, US food and drug administration. Own illustration

However, shorter and smaller trials pose a challenge to correctly evaluate a drug’s risks and benefits. Drugs with fast track or accelerated approval are associated with more unrecognized adverse events and post-marketing safety revisions, e.g., withdrawals or warnings [12–15]. Large trials with a long follow-up are necessary to capture a drug’s most common side effects and observe all risks associated with administering the drug to humans. Similar to drugs approved under the fast track program, the FDA may require post-marketing trials for drugs approved under the accelerated approval program. However, several studies find phase 4 trials to be delayed or not event initiated [18, 19, 89]. In some cases, phase 4 trials even found drugs to be ineffective or harmful, resulting in their market withdrawal [90]. Nevertheless, clinical guidelines are insufficiently updated after post-marketing trial result announcements [90]. The post-marketing trials themselves are subject to debate. Only a few phase 4 trials report clinical endpoint measures [91, 92] and their duration is no longer than those of the pivotal trial [93]. Some authors, therefore, propose reforms to the accelerated approval pathway to strengthen the requirements for and clinical validity of post-marketing trials [94–97] and explore the feasibility of alternative ways to measure a drug’s efficacy and safety post-approval, e.g., real-world evidence^13^ (RWE) [98]. Nonetheless, Chambers et al. reported higher median incremental QALY gains for drugs with accelerated approval (0.370 vs. 0.031, *p* = 0.019) based on a study of 135 indications approved between 1999 and 2012 [77]. In contrast, Hwang et al. could not confirm that drugs with accelerated approval have a greater therapeutic value [43]. From 2003 until 2022, an average of 15% of drugs received accelerated approval (Fig. 5).

Furthermore, the accelerated approval of drugs poses a challenge to insurers and payers [99, 100]. Although there is no difference in the magnitude of prices for drugs with accelerated relative to standard approval, their uncertain evidence bases pose a major challenge for insurers and payers [48]. Without clinical outcome data, insurers and payers cannot adequately measure a drug’s incremental value compared to the treatment alternatives and thereby struggle to decide on the drug’s price, reimbursement, and coverage [101, 102]. “For public insurers, accelerated approval thus becomes not only a pathway for a new product to enter the market but also a mandate to pay high prices for an unproven therapy” [100]. Consequently, European countries established a variety of tools, e.g., performance- and financial-based managed entry agreements (MEAs) with clinical and economic restrictions, to account for this uncertainty in clinical benefits and cost-effectiveness [103–106]. In contrast, the pricing and reimbursement of drugs with an uncertain safety and efficacy profile remain largely unregulated in the US. The free US market seems to only account for the withdrawal of indications with accelerated approval with a price discount of -24% [48]. Although accelerated approval drugs only consume a fraction of Medicare resources, expenditure on them continues to grow [26, 107]. Scholars are especially concerned about spending money on drugs without a proven benefit or on drugs that turn out to be ineffective [27, 108]. Consequently, authors proposed to raise Medicare rebates for drugs with accelerated approval to contain pharmaceutical spending in the US [99, 100].

## Priority review

In the same year as the accelerated approval pathway was passed, in 1992, the US also introduced priority review.^14^ Priority review substituted a more complex system under which the FDA prioritized and allocated more resources to NDA according to their therapeutic gain—classified into three groups (A, B, or C) [8]. Under the new Prescription Drug User Fee Act (PDUFA), these three categories were simplified into two categories: priority or standard review. Thereby, the FDA intended to expedite the regulatory review period, not the drug development process, of drugs that offer a significant therapeutic improvement [2]. The FDA states that a significant therapeutic improvement constitutes “evidence of increased effectiveness”, “elimination or substantial reduction of a treatment-limiting drug reaction”, improved patient compliance leading to better outcomes, or “evidence of safety and effectiveness in a new subpopulation” [2]. Standard NDAs are reviewed in 10 months (reduced from 12 months in 2002), while priority NDAs ought to be reviewed in 6 months by the FDA. Unlike all other special designations, the FDA decides on the review pathway for every NDA and supplementary indication it receives. For all other designations, the pharmaceutical company must submit an additional application for each special designation (Table 1).

**Table 1:** An overview of the FDA’s special review pathways and designations

	Orphan	Fast track	Accelerated approval	Priority review	Breakthrough therapy
Year	1983	1988	1992	1992	2012
Nature of the program	Designation	Designation	Approval pathway	Designation	Designation
Referencse	Orphan drug act of 1983	Section 506(b) of the FD&C act, as added by Section 112 of the food and drug administration modernization act of 1997 (FDAMA) and amended by Section 901 of the food and drug administration safety and innovation act of 2012 (FDASIA)	21 CFR part 314, subpart H 21 CFR part 601, subpart E Section 506(c) of the FD&C act, as amended by Section 901 of FDASIA	Prescription drug user fee act of 1992	Section 506(a) of the FD&C Act, as added by Section 902 of FDASIA
Eligibility	A drug that treats a disease that (A) Affects less than 200,000 persons in the US, OR (B) Affects more than 200,000 in the US and for which there is no reasonable expectation that the cost of developing and making available in the US a drug for such disease or condition will recover from sales in the US of such drug. Determinations under the preceding sentence with respect to any drug shall be made on the basis of the facts and circumstances as of the date the request for designation of the drug under this subsection is made	A drug that is intended to treat a serious condition AND nonclinical or clinical data demonstrate the potential to address unmet medical need OR A drug that has been designated as a qualified infectious disease product	A drug that treats a serious condition AND generally provides a meaningful advantage over available therapies AND demonstrates an effect on a surrogate endpoint that is reasonably likely to predict clinical benefit or on a clinical endpoint that can be measured earlier than irreversible morbidity or mortality (IMM) that is reasonably likely to predict an effect on IMM or other clinical benefit (i.e., an intermediate clinical endpoint)	An application (original or efficacy supplement) for a drug that treats a serious condition AND, if approved, would provide a significant improvement in safety or effectiveness OR Any supplement that proposes a labeling change pursuant to a report on a pediatric study under 505A OR An application for a drug that has been designated as a qualified infectious disease product OR Any application or supplement for a drug submitted with a priority review voucher	A drug that is intended to treat a serious condition AND preliminary clinical evidence indicates that the drug may demonstrate substantial improvement on a clinically significant endpoint(s) over available therapies
Features	Tax credit (25%)^a^ R&D grants User fee waiver More collaboration with and guidance by the FDA Extended period of market exclusivity (7 years)^b^	More frequent communication with the FDA Approval based on phase 2 trial Potential for rolling review	Approval based on surrogate or intermediate clinical^c^ endpoint	The FDA must review the NDA within 6 instead of 10 months^d^	All fast track benefits Communication with the FDA as soon as phase 1 Meetings with senior FDA staff Cross-disciplinary project lead Adaptive and efficient trial design
Conditions	–	FDA may request a phase 4 trial	FDA may request a phase 4 trial	–	FDA may request a phase 4 trial
Effect on	Drug development and market exclusivity	Trial design and FDA review	Trial design and FDA review	FDA review	Trial design and FDA review

Adapted from [3, 8, 143]

*FDA* US food and drug administration, *NDA* new drug application, *R&D* research and development

^a^The tax credit was reduced from 50 to 25% in 2017

^b^Extended from a market exclusivity period of 5 years

^c^In 2012, congress amended that the FDA may also use the accelerated approval pathway for drugs with an intermediate clinical endpoint

^d^The standard review timelines were reduced from 12 to 10 months in 2002

**Fig. 6:**
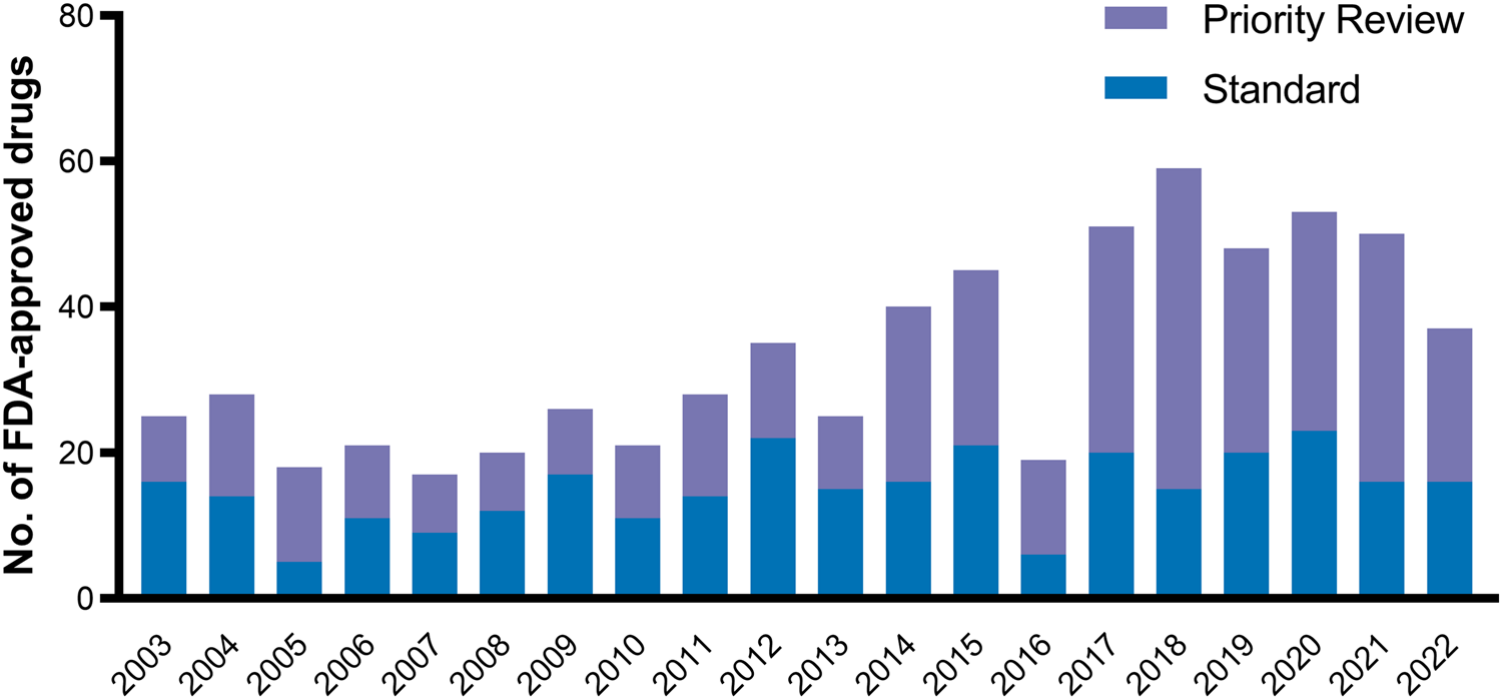
FDA-approved drugs with standard and priority review from 2003 until 2022. *FDA* US food and drug administration. Own illustration

Priority review is the most common special designation, with 34 out of 50 (68%) new drugs benefitting from it in 2021[109]. On average, 55% of new drugs benefit from priority review (Fig. 6). As expected, several studies consequently found a significant reduction in FDA review times over the past decades [3, 110]. However, as previously mentioned, shorter review times were found to be associated with more post-approval safety revisions. Berlin found that drugs approved under the priority review were twice as likely to have labeling revisions after FDA approval than those approved under standard review [111]. Indications with priority review were shown to offer a greater median health gain to patients than those approved under standard review (0.175 QALYs vs. 0.007, *p* < 0.001), referring to the aforementioned study from Chambers et al. [77]. Accordingly, the aforementioned study by Hwang et al. reported a fourfold greater likelihood for drugs with priority review to have a high therapeutic value [43].

## Breakthrough therapy designation

**Fig. 7:**
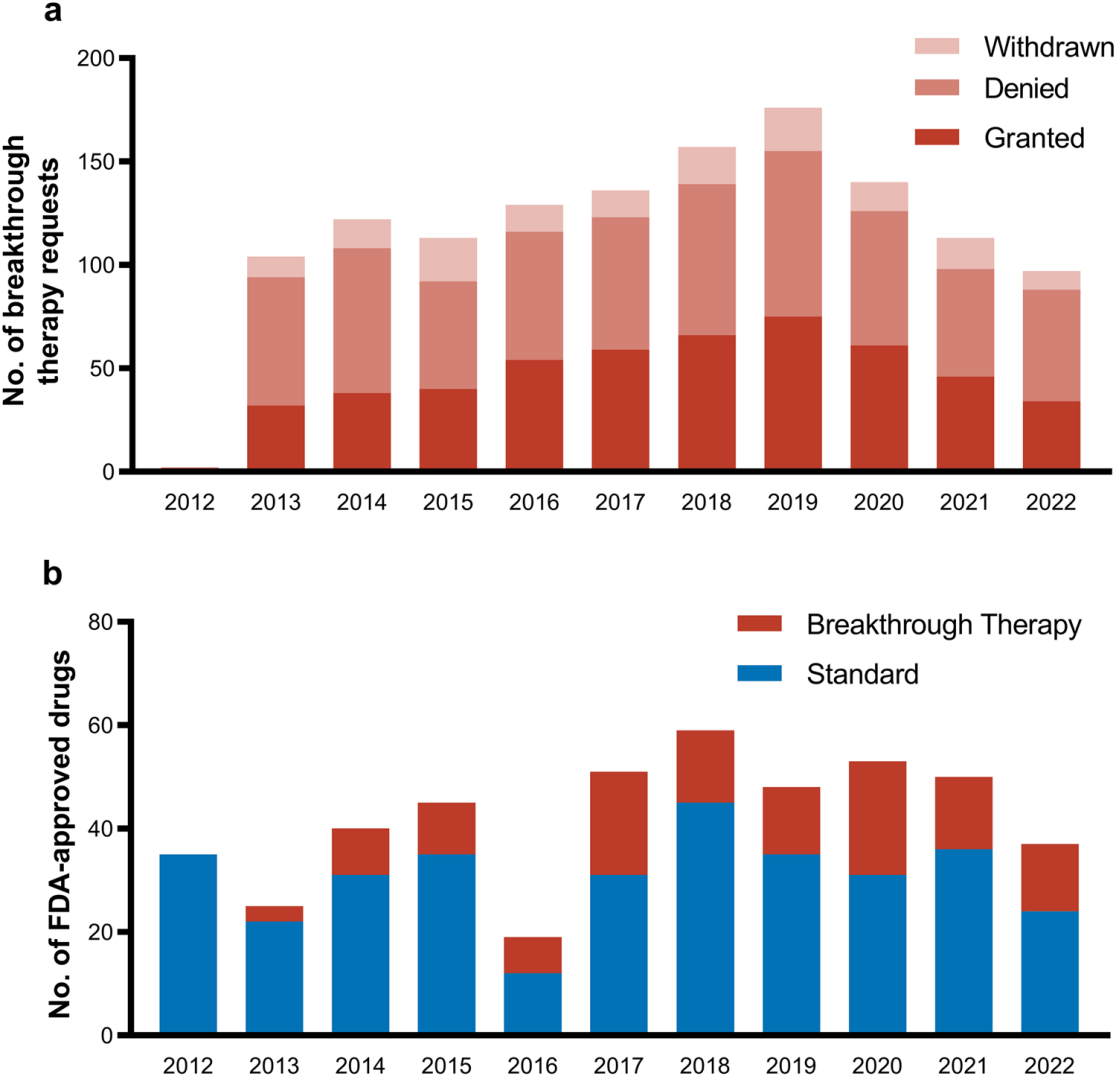
Breakthrough therapy designation requests (**a**) and FDA approvals (**b**) from 2012 until 2022. FDA, US Food and Drug Administration. Own illustration

In 2012, the US Congress introduced a new FDA review pathway as part of the Food and Drug Administration Safety and Innovation Act: The Breakthrough Therapy program.^15^ This program was signed into law to expedite the development and regulatory approval of drugs with a preliminary large benefit in treating a serious or life-threatening disease [2]. Although previous special review pathways have a similar aim, the intentions, benefits, and conditions for the breakthrough designation differ. Congress argued that recent biotechnological discoveries, e.g., next-generation sequencing, gene therapies, or cellular therapies, and advancements in the way drugs are developed, e.g., leveraging precision medicine or RWE, need to be reflected in the regulatory approval process. According to this argument, precisely the efficacy of innovative drugs, such as targeted or gene therapies, can already be observed in phase 2 trials, and therefore large-scale Randomized controlled trials (RCTs) are unethical and unnecessary for regulatory approval [11]. In this context, the 21^st^ Century Cure Act (2016) even encouraged the FDA to use nontraditional clinical trials, new data analysis methods, RWE, observational studies, biomarkers, and surrogate endpoints for the basis of drug approval, sparking further controversy about the evidence supporting new drugs’ safety and efficacy [114, 115]. The program offers earlier (already during phase 1) and more frequent meetings with senior FDA personnel to guide an efficient drug development and regulatory review process. In addition, drugs designated under the breakthrough program receive all of the fast track program’s benefits [2]. As previously stated, the condition for receiving the designation is a large preliminary benefit. In contrast to other programs, this benefit may be observed based on an established surrogate endpoint, a surrogated endpoint that is likely to predict the clinical outcome, reasonable biomarkers, or an improved safety profile with efficacy similar to the standard of care [2]. Therefore, the breakthrough process offers more flexibility to pharmaceutical companies and the FDA in designing clinical trials and measuring clinical benefits. Following its introduction in 2012, the program quickly gained in popularity. Until the end of 2022, the FDA received a total of 1,289 breakthrough therapy requests of which 506 (39%) were granted (Fig. 7a). This led to the approval of 125 new breakthrough-designated drugs (Fig. 7b).

A variety of studies compared the clinical trial characteristics, endpoints, timelines, efficacy, and safety of breakthrough and non-breakthrough therapy-designated drugs. Most studies observed that breakthrough drugs are more frequently approved based on non-randomized single-arm studies of open-label blinding [20, 21, 110, 116, 117]. This is especially concerning given that non-RCTs are associated with a higher frequency of unrecognized serious adverse events, and therefore these drugs’ label often needs to be revised after FDA approval [118] However, Pregelj et al. doubt that this difference in clinical trial characteristics is attributable to the breakthrough designation [119]. They found no significant difference between trials that were already started before the introduction and receipt of the breakthrough status compared to those that commenced after the new legislature. Nonetheless, they observed that the study duration was reduced by 8 months for trials conducted after the new designation. This result indicates that “designations granted early in clinical development may reduce trial time by influencing aspects of clinical programs other than design characteristics, such as timelines for FDA responses”[119].

Consequently, the clinical development time, defined as the period between IND and FDA approval, was observed to be 2–3 years faster for breakthrough compared to non-breakthrough cancer drugs [21, 117, 120]. Breakthrough drugs and indications were more likely to be first-in-class agents, treat novel diseases, act via an innovative mechanism of action, and displayed a tendency to be of novel drug types, e.g., antibody–drug conjugates, gene therapies, cell therapies, and radionuclides [117]. No difference in the number of adverse events or deaths could be observed between breakthrough- and non-breakthrough-designated cancer drugs [21, 120].

There remains an ongoing debate surrounding breakthrough drugs’ efficacy. In a sample of nine cancer drugs, Kern found a high clinical benefit, in terms of PFS and objective response for drugs, for drugs with the breakthrough designation and accelerated approval [22]. In contrast, Hwang et al. found no significant difference in PFS and response rates between breakthrough- and non-breakthrough-designated drugs based on a sample of 58 new FDA-approved drugs between 2012 and 2017 [21]. Similarly, Molto et al.’s analysis of 52 cancer drugs approved across 96 indications between 2012 and 2017 offers inconclusive evidence about breakthrough drug’s efficacy [20]. Only two out of four clinical benefit frameworks^16^ showed a significantly higher efficacy for breakthrough drugs. In 2018, Herink et al., therefore, concluded that breakthrough drugs’ quality of evidence is heterogeneous; therefore, healthcare professionals must be aware that “much remains unknown about the safety and efficacy of many agents approved through the breakthrough therapy designation” [23]. However, all of the above-referenced studies are not only limited in sample size but also focus on the first 5 years of the breakthrough therapy designation (2012–2017). Possibly the breakthrough therapy’s effect may only be apparent several years after it has been passed. In the largest study of breakthrough drugs to date, 355 breakthrough and non-breakthrough cancer indications with FDA approval were compared from 2012 until 2022 [117]. In this study, breakthrough indications were associated with a lower likelihood of death than non-breakthrough indications (hazard ratio: 0.69 vs. 0.74, *p* = 0.031) and offered significantly greater improvements in median overall survival (4.8 vs. 3.2 months, *p* = 0.004). Accordingly, Hwang et al. showed that breakthrough drugs are four times more likely to be of high therapeutic value [43].

Although there is no conclusive evidence that breakthrough-designated drugs are more innovative, safer, or more effective, consumers and physicians hold very positive connotations associated with the term “*breakthrough”* [121–123]. The term “*breakthrough*” implies that these drugs are a major scientific disruption, ultimately inducing misleading unwarranted optimism. Compared to a facts-only description, the term breakthrough convinces a higher percentage of consumers to believe that the drug is very or completely effective (11% vs. 25%, *p* = 0.001) and supported by strong or very strong evidence (43% vs. 63%, *p* = 0.003) [121]. When given the choice between two drugs with the same safety, efficacy, evidence, and cost, 94% of physicians opted to prescribe the drug with the breakthrough designation [122]. Consequently, there is a debate about the “laudatory labels that promote the use of new drugs that frequently offer limited additional benefits” [11].

**Fig. 8:**
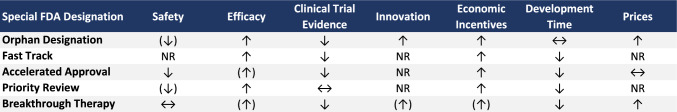
Impact of the FDA's special designations on the safety, efficacy/clinical benefit, trials, innovativeness, economic incentives, development times, and pricing of new drugs. *FDA*, US Food and Drug Administration; *NR*, not reported. The figure illustrates each of the five special designation’s implications on the seven examined dimensions: safety, efficacy/clinical benefit, clinical trial evidence, innovation, economic incentives, development time, and price. Own illustration

Even though breakthrough drugs are approved based on weaker clinical trial evidence and their efficacy is not superior, monthly treatment costs were approximately $16,000 higher for breakthrough compared to non-breakthrough cancer drugs ($38,971 vs. $22,591, *p* = 0.0592) [20, 117]. Consequently, the price premium associated with the designation could be perceived as a positive signal to investors. However, Hoffmann et al. found no long-term excess returns of publicly listed companies developing a drug that recently received a breakthrough designation [124]. Figure 8 summarizes the impact of the FDA's special designations on the safety, efficacy/clinical benefit, trials, innovativeness, economic incentives, development times, and pricing of new drugs.

## Special designation in the EU (EMA) and the US (FDA)

**Fig. 9:**
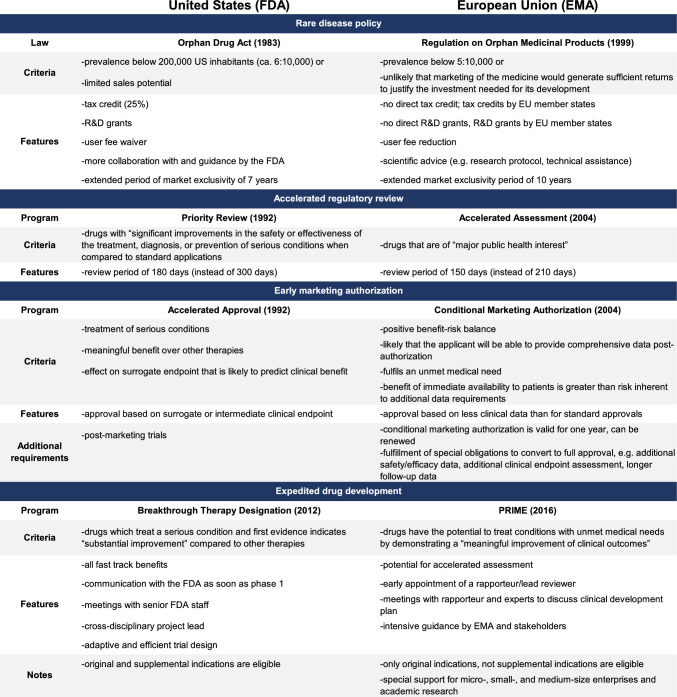
Comparison of special designations and review programs in the US (FDA) and the EU (EMA). *EMA* European medicines agency; *FDA* US food and drug administration; *PRIME* priority medicines, *R&D* research and development. Own illustration

Analog the Orphan Drug Act of 1983, the EU passed the Regulation on Orphan Medicinal Products in 1999 (Fig. 9) [125]. Drugs that treat rare diseases with a prevalence below 5 in 10,000 EU inhabitants or drugs with limited sales potential in relation to their R&D spending are eligible to receive the designation. The EMA orphan designation provides a marketing exclusivity period of 10 years (FDA: 7 years), a user fee reduction that is higher for micro-, small-, and medium-sized enterprises (FDA: user fee waiver), and scientific advice. Tax credits and additional research grants are not provided by the EMA but are funded by national drug development programs of the EU member states. A recent study highlighted that although the eligibility criteria for the FDA and EMA orphan designation are fairly similar, the implementation between both agencies differs [63]. Less than half of drugs with FDA orphan designation also received the EMA orphan designation. This is particularly interesting because the FDA regards biomarker-based subgroups of common diseases as distinct orphan indications, whereas the EMA only rarely agrees with this logic. Supporting the EMA’s interpretation of rare disease policy, recent studies showed that biomarker-based subgroups of common diseases are more similar to common than truly rare diseases, and henceforth “ill-suited” for the orphan designation [38, 62]. Furthermore, the EMA’s Orphan Medicinal Products law has a “*clawback*” clause (that has never been used) which permits the EMA to limit the period of market exclusivity from 10 to 6 years for high-grossing drugs [126]. This “*clawback*” clause could be especially useful to limit expenditure on top-selling partial orphan drugs, e.g., drugs that are commercialized for orphan and non-orphan indications [69, 71].

Similar to the FDA’s priority review program, the EMA’s accelerated assessment program reduces the regulatory review time by 210 to 150 days (Fig. 9) [127, 128]. Indications that represent a “major public health interest” are eligible for accelerated assessment. However, there is no clear definition of “major public health interest” [129]. The pharmaceutical company itself must submit a justification that entails a description of the treated disease’s unmet medical need, current methods to prevent, diagnose, and treat the disease, and the drug’s efficacy and evidentiary basis in filling these unmet medical needs. In contrast to the FDA’s priority review program, pharmaceutical sponsors must actively request accelerated assessment 2–3 months before submitting the marketing authorization application.

Comparable to the FDA’s accelerated approval program, the EMA introduced the conditional approval program in 2004 (Fig. 9) [82, 130]. However, there are succinct differences between these programs [85]. Drugs with (1) a positive benefit–risk ratio, (2) a great likelihood that the applicant will be able to provide comprehensive data post-authorization, (3) fulfillment of an unmet medical need, and (4) a benefit of immediate availability to patients that is greater than the risk inherent to additional data requirements are eligible for conditional approval. For these drugs, the EMA may grant conditional approval based on less clinical data than standard approvals. In contrast to the FDA’s accelerated approval, the EMA’s conditional approval is, therefore, not subject to a surrogate endpoint. Drug sponsors must fulfill special obligations to renew the conditional approval every year and/or convert to full approval. These special obligations are defined by the EMA and may, for example, include additional safety and efficacy data, longer follow-up data, and/or the assessment of additional endpoints. On average, conditional approvals are converted to full approvals after 4 years [130]. As a result of these narrow eligibility criteria and strict post-approval requirements, fewer conditional/accelerated approvals are granted to fewer drugs by the EMA and FDA [43, 131]. However, these strict post-approval requirements also resulted in fewer post-approval withdrawals for the conditional relative to the accelerated approval program [129].

Similar to the FDA’s breakthrough therapy program, the EMA introduced the priority medicines (PRIME) program in 2016 (Fig. 9) [132]. The program was initiated to support the development of drugs that have the potential to treat conditions with unmet medical needs by demonstrating a “meaningful improvement of clinical outcomes.” The benefits of this program include an early appointment of a rapporteur/lead reviewer, iterative and early meetings with the rapporteur and disease experts to discuss the clinical development plan and seek scientific advice, and overall intensive guidance by the EMA and key stakeholders. PRIME drugs are potentially eligible to receive an accelerated assessment. In contrast to the FDA’s breakthrough therapy designation, the PRIME program can only be granted to original, yet not supplemental, indications. Furthermore, micro-, small-, and medium-sized enterprises, and academic research institutes may receive earlier PRIME guidance based on promising pre-clinical data. Although there is no separate fast track designation in the EU, the PRIME program has certain features that resemble the FDA’s breakthrough therapy and fast track programs. [129].

## Limitations

There are several limitations inherent to our article. First, we focused on the FDA’s special designations and review pathways given that the US represents the largest pharmaceutical market, is the most important country for drug development, and the FDA is generally the first agency that approves new drugs. However, review programs from other regulatory agencies, e.g., EMA, TGA, or HC, may influence the global drug development process. Second, there are other special FDA programs such as the Tropical Disease Priority Review Voucher Program, Rare Pediatric Disease (RPD) Designation and Voucher Programs, Emergency Use Authorization, and Expanded Access (also called “compassionate use”). Third, this is the first narrative review to assess the FDA’s special approval pathways and designations regarding their safety, efficacy/clinical benefit, clinical trials, innovation, economic incentives, development timelines, and price. Building on our findings, future researchers should conduct a systematic review on this topic. Fourth, many studies referenced in this article examined cancer drugs. Although oncology is the largest therapeutic area in drug development, the implications of special FDA designations and approval pathways on the examined dimensions may differ. Fourth, referenced articles typically focus on a single special FDA designation. However, each drug and indication may receive multiple special designations. The influence of multiple, “stacked”, special FDA designations is only scarcely reported and discussed in scientific literature [144]. Future studies should evaluate the impact of the cumulative number and types of special designations on drug development.

## Conclusion

The FDA’s special designations incentivize and facilitate the expedited development of drugs for rare and severe medical conditions. Over the past decades, these designations provided pharmaceutical companies with more flexibility in conducting clinical trials to provide patients with timely access to promising new drugs. Although these programs progressively lowered the FDA’s evidentiary standard for a drug’s safety and efficacy requirements, patients pay a premium for drugs whose “laudatory labels” provide high profits for pharmaceutical corporations and investors. Nonetheless, the majority of reviewed studies found that drugs with special designations provide a higher clinical benefit to patients than those without special designations. Yet, this greater benefit is not proportional to the substantially higher prices that are demanded for special-designated drugs. Instead of creating more special review programs for potentially unsafe, yet expensive drugs, politicians should reshape existing pharmaceutical policies to truly incentivize the development and approval of safe, effective, innovative, and affordable drugs that are tested in robust RCTs.

## Data Availability

Not applicable.

## Footnotes

1 These special designations are interchangeably referred to as special review pathways, procedures, or processes.

2 Unmet medical need is defined by three factors: (1) the quantity and quality of available therapeutic options, (2) the burden of disease, and (3) the disease incidence [1].

3 There are a variety of other programs which aim to incentivize the development of and grant earlier access to new treatments, e.g., Priority Review Vouchers [4], Emergency Use Authorization [5], Expanded Access Program (also called compassionate use) [6, 7]. The presented five are the most common and widely used special review programs which apply across all therapeutic areas [3, 8].

4 One hundred twenty-four breakthrough designations relative to a total four hundred twenty-seven drugs that received approval after the breakthrough therapy designation were signed into law in 2012.

5 The FDA’s obligation and authority to review and exclude drugs from the market that are not effective were legally established in the Kefauver–Harris Amendments (1962).

6 A company must apply for additional regulatory approval in other jurisdictions if it seeks to commercialize the new drug outside of the US. Regulatory agencies around the globe are responsible for their respective jurisdiction, e.g., the European Medicines Agency (EMA) is responsible for the EU, Health Canada (HC) for Canada, and the Therapeutic Goods Administration for Australia.

7 Under the ODA, a rare disease is defined by a prevalence below 200,000 patients per year in the US, equivalent to a prevalence rate of about 6 in 10,000.

8 Most countries around the world followed the US example and incentivized the development and market availability for drugs treating rare diseases [30, 31]. England and Scotland even introduced an ultra-orphan designation for diseases with an incidence rate below 0.2 per 10,000. However, substantial difference in the magnitude of orphan drug policies exist, especially between high- and low-income countries [32].

9 The tax credit was reduced from 50 to 25% in 2017 [11].

10 The ICER is a measure of an intervention’s benefit and costs relative to a comparator, usually the gold standard of care. Health technology assessment (HTA) agencies, such as England’s National Institute for Health and Care Excellence (NICE), evaluate a drug’s efficacy, safety, and proposed pricing based on this ratio. Some countries, such as England, set a willingness-to-pay (WTP) threshold for a drug’s ICER. An ICER above this threshold is commonly deemed not cost-effective, and therefore will not be reimbursed by insurers.

11 Discrepancies between success rate estimates are mainly attributable to differences in the underlying database, time period, and methodology (phase-by-phase vs. path-by-path) used for the calculation.

12 The EU, Canada, and Australia quickly followed and introduced the conditional marketing authorization, the notice of compliance with conditions, and the provisional approval of drugs, respectively [82–84]. However, Mehta et al. pointed out that there are peculiarities associated with each of these programs [85].

13 RWE refers to clinical data derived from electronic health records, claims and billing records, drug registries, mobile apps, and the like.

14 Again, other countries followed suit. The EU introduced the accelerated assessment in 2005. Similar to the US, Canada and Australia both named their accelerated review pathway *priority review*.

15 Other countries quickly followed the US in introducing new drug approval pathways: Japan passed the Sakigake designation in 2015 and the EU the Priority Medicines Scheme (PRIME) in 2016 [112, 113].

16 They evaluated a drug’s clinical benefit based on four distinct scoring systems: (1) American Society of Clinical Oncology Value Framework (ASCO-VF), (2) American Society of Clinical Oncology Cancer Research Committee (ASCO-CRC), (3) European Society for Medical Oncology Magnitude of Clinical Benefit Scale (ESMO-MCBS), (4) National Comprehensive Cancer Network (NCCN) Evidence Blocks.

## Declarations

### Provenance and peer review

Not commissioned; externally peer-reviewed.

### Patient and public involvement

No patients were involved in the conduct of this study.

### Transparency statement

D.T.M. (the manuscript's guarantor) affirms that the manuscript is an honest, accurate, and transparent account of the study being reported; that no important aspects of the study have been omitted; and that any discrepancies from the study as originally planned have been explained.
